# Insertion of orthodontic temporary anchorage devices with free gingival grafting for phenotype modification of the peri-implant mucosa

**DOI:** 10.1016/j.jobcr.2023.09.005

**Published:** 2023-09-29

**Authors:** Oscar Maldonado Molina

**Affiliations:** a Postgraduate Orthodontic Program, Universidad de San Carlos de Guatemala, Guatemala City, Guatemala; b Postgraduate Orthodontic Program, Universidad Intercontinental, México City, Mexico

**Keywords:** Orthodontics, TADS, Orthodontic biology, Phenotype modification, TAD grafting, Orthodontic mini implants

## Abstract

**Background:**

Mini Implants are widely used in contemporary orthodontics, they provide skeletal anchorage even in non-compliant patients, facilitate orthodontic tooth movement, are easy to place and are relatively inexpensive. Their failure is multifactorial, and the quality of the soft tissue can present a risk limitation for the insertion of TADS. Orthodontic Mini Implants inserted in keratinized gingiva present fewer tissue-related complications and higher survival rate, than those inserted in non-keratinized mucosa. The purpose of this report is to present and describe this novel technique to modify and enhance the peri-implant mucosa of Orthodontic Mini Implants inserted in nonkeratinized gingiva.

**Methods:**

A free gingival graft was harvested from the palate in combination with a buccal recipient site preparation in the alveolar mucosa and a TAD insertion procedure.

**Results:**

After twenty-one days of healing, graft integration was observed. One hundred and eighty days after insertion and twelve weeks of loading, none to mild signs of clinical inflammation were documented, and the patient reported no pain or discomfort.

**Conclusion:**

Within the limitations of this report, free gingival grafting for phenotype modification of the peri-implant mucosa, can benefit patients who need insertion of orthodontic mini-implants in non-keratinized mucosa for orthodontic tooth movement.

## Graphical abstract

Orthodontics, adult treatment in orthodontics, anchorage, orthodontic biology, periodontics.

**Figure:**
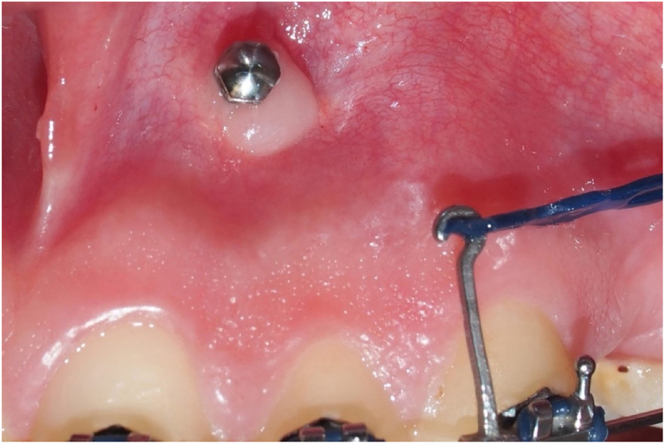

## 1 Introduction

Since the first clinical reports of skeletal anchorage with implants,^1^^,^^2^ Orthodontic Temporary Anchorage devices (TADS) have broadened the scope of orthodontic treatment.^3^ Mini implants inserted in keratinized gingiva present higher success rates, and patients present fewer tissue-related complications.^4^^,^^5^

For insertion site selection, TADS should be placed in the safest anatomical position, and the presence of keratinized gingiva is preferred. Considering a 1.5 mm diameter mini screw, a minimum of 3 mm space between the roots of neighboring teeth is recommended. The interradicular distance is greater toward the apices. To reduce the possibility of contacting a root, a 30–60° apical angulation should be used.^6^^,^^7^ Orthodontic Mini Implants placed in movable alveolar mucosa could present a risk of tissue irritation. The insertion zone of opportunity has been described as 2 mm extending apical from the mucogingival junction, where the mucosa has virtually no mobility. But root proximity could still be a problem, requiring placing the mini screws more apical to the Muco-Gingival Junction, where the mucosa is thinner and more mobile and the mini screws present higher incidence of tissue overgrowth, inflammation, micro-tears and ulcerations that cause pain or discomfort to the patient.^8^^,^^9^

Thin phenotype and inadequate width of keratinized gingiva (<2 mm) may be a significant risk indicator for peri-implant mucositis, peri-implantitis and pain/discomfort during brushing.^10^

Palatal gingival grafts were first described by Bjorn in 1963. Since then, free gingival grafts and connective tissue grafts to increase the width of the keratinized gingiva or augment the gingival thickness have been reported with high success rates.^11^, ^12^, ^13^

Regarding the potential benefits of phenotype modification therapy on gingival health, biotype defines a specific genetic trait, whereas phenotype is a multifactorial combination of genetic traits and environmental factors. Gingival phenotype is site specific and contain components that can change over time depending on environmental factors. Phenotype modification therapy can create a more favorable environment for the prevention of disease and the maintenance of periodontal health.^14^

The potential benefits of phenotype modification for patients receiving orthodontic treatment include enhanced periodontal health, increased stability of orthodontic outcomes, reduced periodontal complications, shortened orthodontic treatment time, optimal periodontal and orthodontic outcomes, expanded opportunities and increased boundaries for treating malocclusions, reduced need for extractions and orthodontic camouflage, and increased limits for arch expansion.^14^

Orthodontic Mini Implants were inserted with free gingival grafting to modify and enhance the peri implant mucosa from thin and non-keratinized to thick and keratinized at the insertion site. Extending the insertion zone of opportunity, providing comfort to the patient, preventing soft tissue-related complications, reducing the risk of contacting the roots of neighboring teeth and being able to apply vectors of force closer to the center of resistance.^15^

**Fig. 1:**
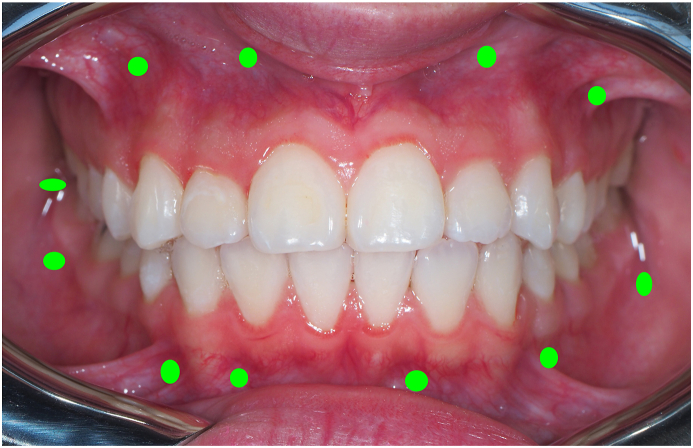
Possible tad grafting sites in non-keratinized mucosa.

TAD Grafting can benefit patients with vestibular interradicular TADS inserted in non-keratinized alveolar mucosa, extra radicular TADS in supra-apical o subapical insertion, mandibular retromolar area and posterior mandibular buccal shelf (as shown in Fig. 1).

## 2 Material and methods

From a group of thirty-six orthodontic patients diagnosed for vestibular inter radicular TAD insertion in private practice. Four patients, who did not have enough inter radicular space in keratinized gingiva for TAD insertion, were selected to receive a free gingival graft at the time of insertion. Two patients have been included in this report (6 Mini Implants, three of them with free gingival grafting) that have completed at least six-month observational period after TAD insertion.

**Fig. 2:**
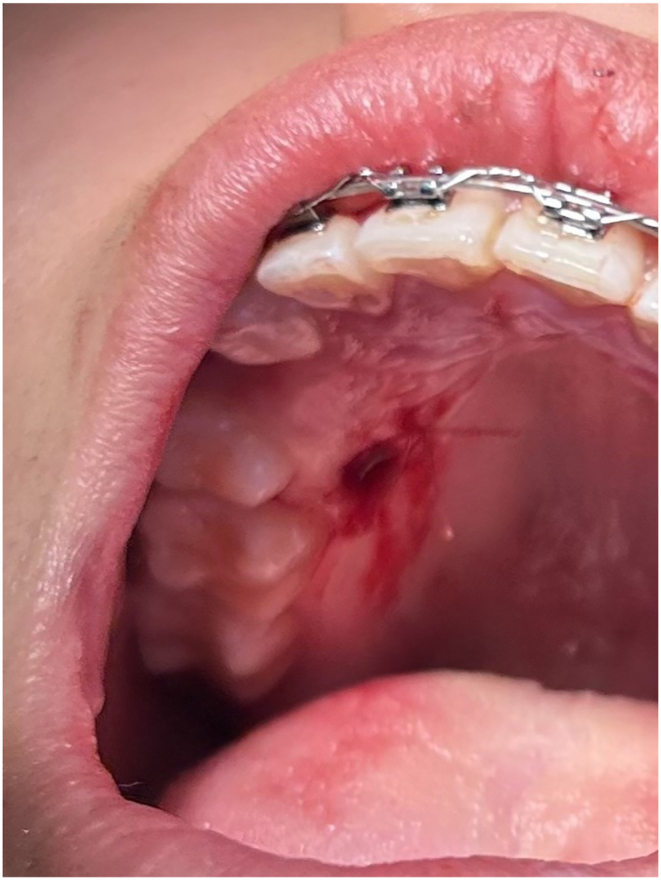
Palatal donor site.

After cleaning with Chlorhexidine and infiltrate lidocaine, a free gingival graft with 2 mm thickness was harvested from the palate, with a 6 mm biopsy tissue punch after de-epithelization. A resorbable wound dressing hemostat was applied for donor site management.^16^ (as shown in Fig. 2).

**Fig. 3:**
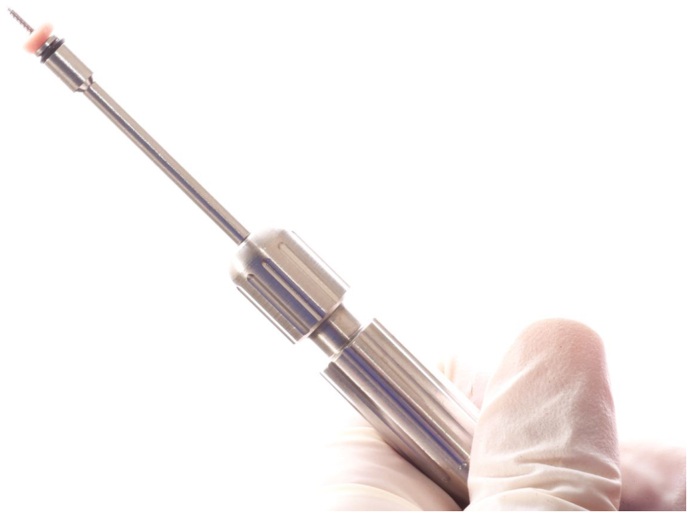
Orthodontic mini implant with gingival graft.

With the pilot drill attached to the screwdriver a hole was made at the center of the graft, and the Orthodontic Mini Implant was inserted through the graft with the screwdriver all the way to the neck (as shown in Fig. 3).

**Fig. 4:**
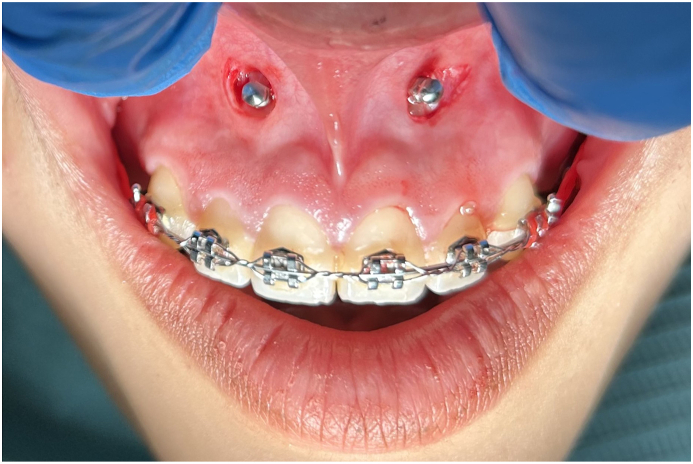
Anterior maxilla, buccal recipient site.

At the recipient site, the buccal mucosa was stretched, and the insertion point was marked with the pilot drill, the tissue around the selected area was de-epithelized with a 3 mm diameter round diamond bur. A self-drilling titanium Orthodontic Mini Implant with 1.5 mm diameter and 8 mm length was inserted until the graft was in contact with the recipient bed, taking care not to over press the graft to prevent necrosis and allow revascularization (as shown in Fig. 4).

The same procedure was performed for the other side of the mouth, if needed. Post operatory instructions, antibiotics, Ibuprofen, and chlorhexidine rinses were prescribed.

## 3 Results

**Fig. 5:**
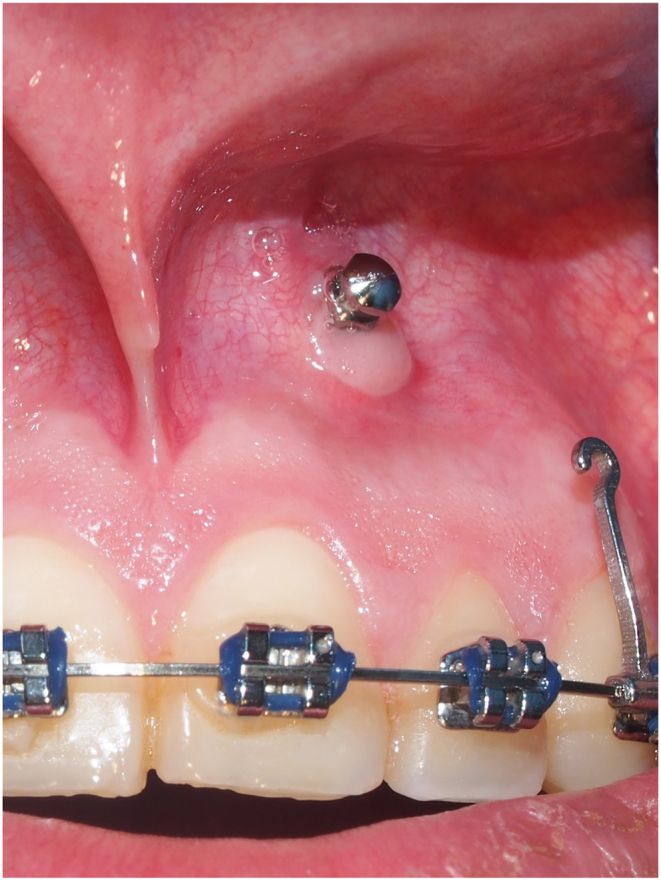
Twenty-one days after insertion, healing and graft integration is observed.

Graft integration was observed after twenty-one days of healing. Pink color, no swelling, no bleeding and firm tissue consistency were documented (as shown in Fig. 5).

After the period of healing, patient was instructed of oral hygiene, and during the next six weeks reported no pain or discomfort and no bleeding after brushing or rinsing.

Sixty days after insertion and four weeks of loading, on visual inspection of peri-implant mucosa, none to mild signs of inflammation were observed. Phenotype modification of the alveolar mucosa allowed the insertion of Orthodontic Mini Implants apical to the mucogingival junction, reducing the risk of contacting the roots of neighboring teeth.

One hundred and fifty days after TAD insertion and twelve weeks of loading, besides inflammation present in the lip mucosa caused by contact and friction with the head of the mini screw, keratinized peri-implant mucosa remained healthy.

**Fig. 6:**
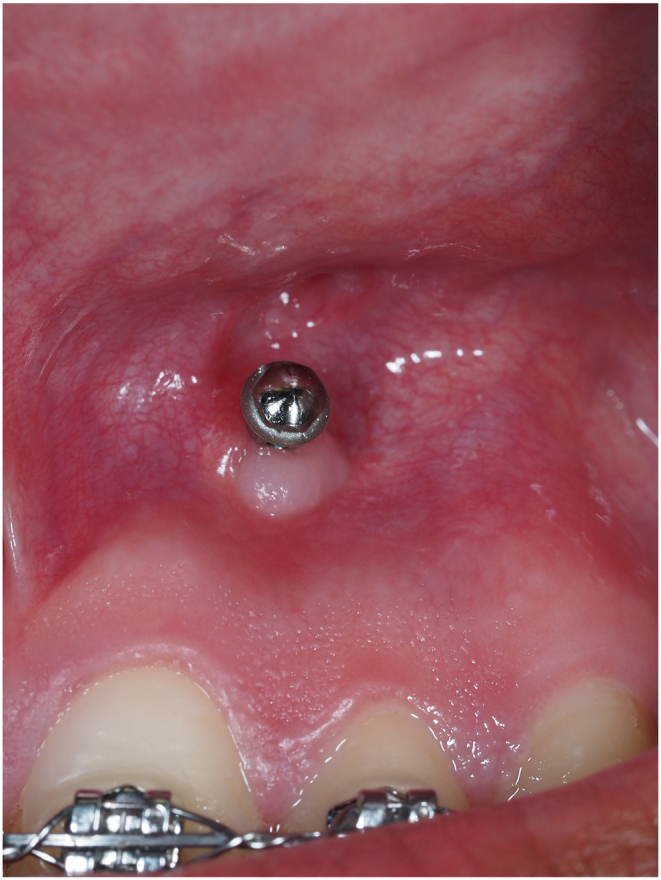
One hundred and eighty days after insertion, peri-implant mucosa remained healthy and prevent tissue overgrowth from the alveolar mucosa.

One hundred and eighty days after TAD insertion, keratinized peri-implant mucosa prevented overgrowth of the movable lining mucosa around the Orthodontic Mini Implants (as shown in Fig. 6).

**Fig. 7:**
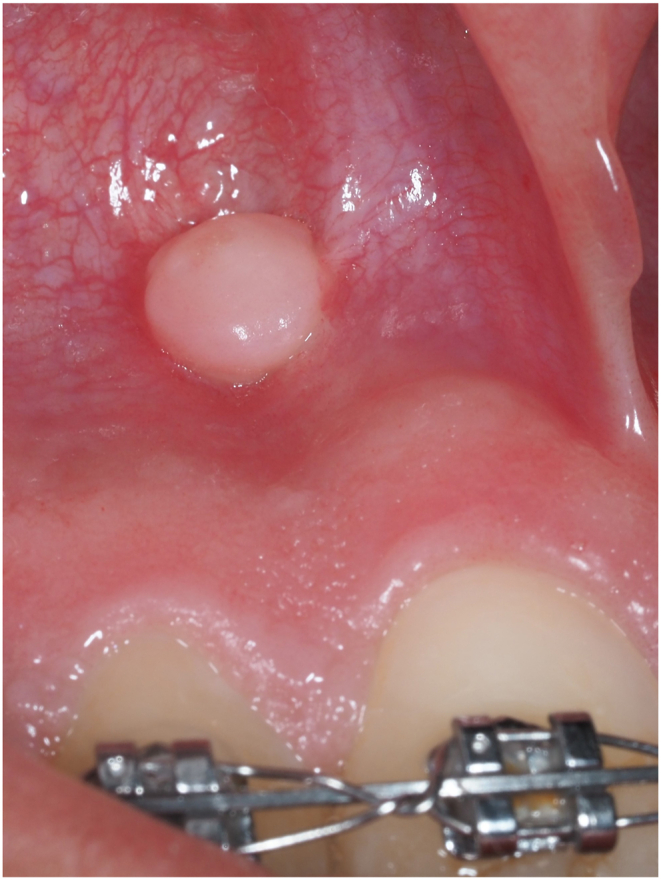
Free gingival graft in the alveolar mucosa of the anterior maxilla, after tad removal.

After tad removal, Free Gingival Graft can be left in situ as in periodontal therapy, or can be removed per patient request, no complications have been observed after tad removal (as shown in Fig. 7).

## 4 Discussion

Houfar et al., reported that the success rate of 387 Orthodontic Mini Implants was primarily affected by the insertion site. Palatal Mini implants were nearly always successful (98.9%), while buccal Mini implants were clearly less successful (71.1%). It appears that buccal interradicular insertion combined with the type of anchorage resulted in a lower survival rate. The presence of keratinized mucosa, the bone density of the anterior palate and the absence of the roots of teeth seem to play an important role in the success rate of paramedian insertion in the anterior palate.^17^

Cheng et al., in a prospective clinical study involving 140 Mini implants, found that the absence of keratinized mucosa around Mini implants significantly increased the risk of infection and failure.^18^ Lai and Chen, in a retrospective study of 266 TADS, reported a survival rate of 96.2% for Orthodontic Mini Implants placed in buccal keratinized mucosa, significantly higher than those placed in oral lining mucosa with a survival rate of 66.7%.^19^ Manni et al., found similar results in a retrospective study of 300 mini-screws, with higher success rates when inserted in attached gingiva or the mucogingival line.^20^

Chen et al., found in a retrospective study of 194 patients with 492 TADS that Inflammation of soft tissue surrounding a TAD and early loading within 3 weeks after insertion were the most significant factors predicting TAD failure.^21^

Lang and Loe, in 1972 wrote about the importance of keratinized gingiva and gingival health, years later in 1995, Lang et al., in an experimental study in monkeys, found that the absence of keratinized mucosa around dental implants increases the susceptibility of the peri-implant region to plaque-induced tissue destruction.^22^^,^^23^

The presence of at least 2 mm of keratinized mucosa around dental implants has a protective effect on the peri-implant tissue condition, facilitates brushing and plaque control, and is associated with less peri-implant inflammation, lower mean alveolar bone loss and improved indices of soft tissue health.^24^

Phenotype modification to enhance the peri-implant mucosa of orthodontic TADS provide a more favorable environment for the prevention of soft tissue complications and the maintenance of the peri-implant health. Specially in non-compliant orthodontic patients.^25^^,^^26^

Orthodontic Mini-Implants are more frequently placed in nonkeratinized mucosa than dental implants. The failure rate reported in the literature for orthodontic TADS placed in keratinized mucosa versus non keratinized mucosa is significant.^27^

Similar techniques have been applied to implant dentistry, using healing screws with connective tissue grafts to augment the gingival volume around implants. The author thinks after online research and PubMed review, that this is the first time that Free Gingival Grafting in combination with a TAD is being applied in orthodontics for TAD insertion in non-keratinized alveolar mucosa.

Four patients met the selection criteria from a group of thirty-six patients, but this is mainly a documentation from the first two cases. The other two cases are in the orthodontic preparation process to receive a TAD. To this date all grafting procedures have been successful.

Excessive pressure to the graft is prevented not inserting the full length of the mini screw, clinician will see when the graft is in contact with the recipient bed and should stop when all the base of the graft has contact with the recipient bed. Blanching of the graft should be prevented with this method, allowing revascularization and preventing necrosis.

Periodontal dressing has not been used to aid for stabilization of the graft, but in the first case, a TAD insertion in the posterior maxilla, the graft did not have contact in all the base, interrupted sutures were added in mesial and distal to stabilize the graft, complete healing was achieved without any further complication. The head of the TAD is wider than the shank, helping to support and stabilize the graft. It is very important to perforate the graft at the center with a pilot drill before inserting the TAD to prevent tearing the graft with the screw.

## 5 Conclusions

Within the limitations of this report of two cases, free gingival grafting for phenotype modification of the peri-implant mucosa, may benefit patients who need the insertion of TADS in nonkeratinized mucosa for orthodontic tooth movement, facilitating oral hygiene and preventing soft tissue-related complications. Additionally, the risk of root contact can be reduced by placing the TADS more apical. Tad insertion with gingival grafting can help clinicians inserting TADS in non-keratinized mucosa for incisor intrusion, molar protraction, mandibular arch retraction, total arch intrusion and distalization. It is important to evaluate the height of the mucogingival junction and the proximity of the roots to determine the need for gingival grafting and select the best site for TAD insertion. Six weeks of healing are necessary for integration and maturation of the graft before loading the TADS.

I have obtained informed consent from patient/s and/or legal guardians, and I have included a separate statement in the manuscript file. - I have obtained informed consent from patient/s and/or legal guardians, and I have included a separate statement with this manuscript file.
